# The Integrated Dyspnea Clinic: An Evaluation of Efficiency

**DOI:** 10.5334/ijic.3983

**Published:** 2018-12-31

**Authors:** Mark V. Rietbroek, Annelies M. Slats, Philippine Kiès, Greetje J. de Grooth, Niels H. Chavannes, Christian Taube, Tobias N. Bonten

**Affiliations:** 1 Department of Pulmonology, Leiden University Medical Center, Leiden, NL; 2 Department of Public Health and Primary Care, Leiden University Medical Center, Leiden, NL; 3 Department of Cardiology, Leiden University Medical Center, Leiden, NL; 4 Department of Pulmonary Medicine, Ruhrlandklinik, West German Lung Center, University Hospital Essen, University Duisburg-Essen, Essen, DE

**Keywords:** dyspnea, cardiorespiratory, health services research, integrated care, outcome evaluation, quality of care

## Abstract

**Introduction:**

Dyspnea is a common complaint and in 70 to 90% the origin is pulmonary or cardiovascular. However, referral to the “wrong” specialism could result in diagnostic- and treatment delay. Integrated care by a cardiologist and a pulmonologist could improve this. The aim of the present study was to evaluate whether integrated care for patients with dyspnea is more efficient and effective than regular care.

**Methods:**

Consecutive patients (n = 235) seen at our dyspnea clinic after June 2014 were included. Two patient groups were compared: 1) patients with an integrated consultation and 2) patients with a non-integrated consultation, who were seen by the cardiologist and the pulmonologist on separate occasions.

**Results:**

The median time until first diagnosis, final diagnosis and time needed for diagnostic workup was shorter for patients evaluated by a integrated consultation compared with patients with a non-integrated consultation for dyspnea (16 days vs. 37 days, p < 0.001; 51 days vs. 78 days, p < 0.001; 35 days vs. 67 days, p < 0.001). There were no significant differences in the majority of diagnostic tests used and final medical conclusions.

**Conclusions:**

Patients with dyspnea evaluated using integrated care were diagnosed almost one month faster than patients in regular care without affecting the type of medical conclusions made. This study supports the start of a dyspnea clinic as an efficient way to provide integrated care to patients with dyspnea.

**Take home message:**

Patients with dyspnea evaluated using integrated care where diagnosed one month faster than patients in regular care.

## Introduction

Dyspnea is a common reason for patients to consult their general practitioner, ranging from 0.9% to 3.9% of all consultations [1,2,3,4]. Patients describe dyspnea as breathlessness, shortness of breath, chest tightness, air hunger, difficult breathing or any variation of these [5,6]. The causes for dyspnea range from a simple common cold to life-threatening causes like myocardial infarction or pulmonary embolism [4,6,7,8]. In the sensation of dyspnea, multiple systems are involved; in a basic model these are the airways, the cardiovascular system, mechanical properties of the chest wall, and the nervous system [5,6]. Apart from physical factors, dyspnea can also be caused or worsened by psychological factors [6]. However, in 70–90% of the patients the origin of dyspnea is pulmonary or cardiovascular [3,7,8,9].

In primary care it is often difficult to distinguish between the different causes of dyspnea. Diagnostics are limited to the medical history, physical examination and minimal additional tests [3,5]. When the cause is unclear or initial treatment does not have sufficient effect, a referral to secondary care might be appropriate [5]. Interestingly, a previous study found there was only 39% concordance between the probable diagnosis on referral and the final diagnosis after complete diagnostic workup in secondary care [10]. In only 33% of the patients referred to cardiology and in 60% of the patients referred to pulmonology the referral diagnosis was correct [10]. Referral to the “wrong” specialism could result in diagnostic- and treatment delay, which results in discomfort and uncertainty for the patient. Importantly, a large proportion (≥30%) of chronic pulmonary patients has- or is at high risk for developing co-existing cardiovascular conditions [11,12]. So, it seems of additional value to evaluate patients presenting with dyspnea in an integrated assessment by a pulmonologist and cardiologist [13]. Such ‘integrated care’ by cardiologists and pulmonologists might be facilitated through a dyspnea clinic, which was started by the Leiden University Medical Center in July 2014. In theory, such a integrated care program results in less diagnostic delay. Although numerous other efficiency frameworks and measures on a patient level or health care system level are available, diagnostic time is an important efficiency measure from the patient perspective and is the focus in this study [14]. Scientific evidence for an effect of a dyspnea clinic on efficiency endpoints is scarce. Only one study assessed the effect of a dyspnea clinic [10], but did not compare dyspnea clinic patients with regular care and therefore cannot be used to determine efficiency of a dyspnea clinic. Therefore, the aim of this study was to evaluate whether this new integrated care strategy is more efficient than regular care.

## Methods

### Study setting

**Figure 1:**
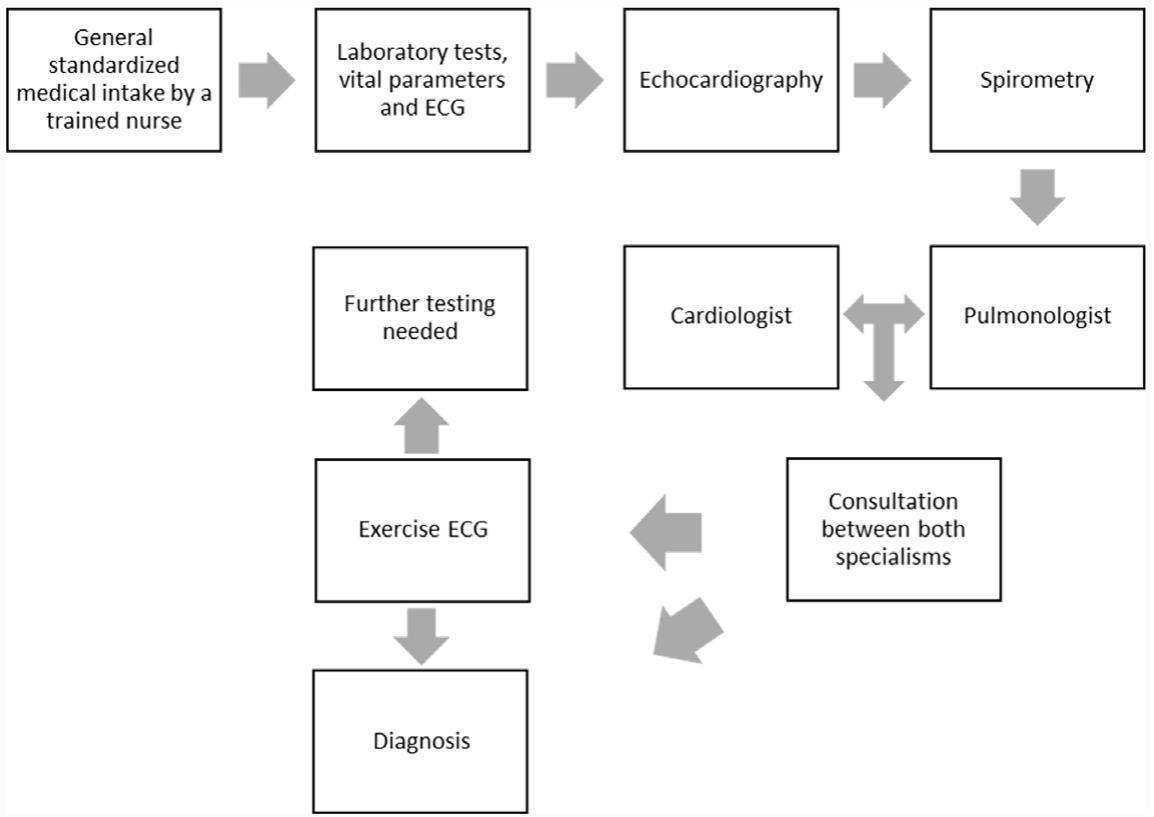
The workflow of the dyspnea clinic.

The integrated care in this study consists of a protocolized and standardized collaboration between the pulmonologist and the cardiologist. The dyspnea clinic is the general name used for this specific form of integrated care. A dyspnea clinic was started in July 2014 by the Leiden University Medical Center in an out-patient clinic situated outside the hospital. Patients referred to this clinic were evaluated in one visit by both the cardiologist and the pulmonologist, of which the workflow is shown in Figure 1.

If extra diagnostics were needed these were performed in the Leiden University Medical Center where the short communication lines between both specialisms remained intact. In addition to this integrated care program, the pulmonologist and cardiologist also examined patients with dyspnea referred to pulmonology or cardiology alone respectively (non- integrated consultation).

### Patient population and data extraction

In this study, we chose to use measures of diagnostic time, number of diagnostics and final diagnoses as efficiency measures. For this, all consecutive patients from June 2014, who were examined by the attending pulmonologist at either the dyspnea clinic or as a non- integrated consultation, were kept in a list in the electronic medical record. Patients who were only seen by a pulmonologist (non-integrated consultation) and not by a cardiologist were excluded, because these patients did not follow the integrated care pathway of the dyspnea clinic. Patients with comorbidities were not excluded. Since the list was composed by the pulmonologist, no patients in our study were seen by a cardiologist only. Additionally, all patients referred after June 2016 were excluded to prevent unfinished diagnostic workup. All relevant data was extracted from the electronic medical records. The following variables were extracted: baseline characteristics (length of patients in centimeters, weight in kilograms, age, gender and smoking status), medical history, medication usage, referral by specialty, referral date, date of consultation (combined and pulmonologist or cardiologist), date of first diagnosis, date of final diagnosis, diagnostics used and conclusions made. For some patients the date of referral was unclear, because they were already under treatment of a cardiologist for a long time. In that case, the date of referral by the cardiologist to the pulmonologist was entered both as the date of referral to- and as the date of consultation with the cardiologist. Only diagnostics performed between the date of referral and the date of final diagnosis were entered into the database. The date of first diagnosis corresponds to the date on which the working diagnosis was stated. The date of final diagnosis corresponds to the date when the final conclusion was made (after all optional supplementary diagnostics). For analysis on outcome of diagnoses, only the final conclusions were used. Multiple conclusions per patient were allowed, because dyspnea can be caused by a combination of conditions. The conclusions ‘deconditioning’ and ‘dysregulated breathing mechanisms’ were concluded when other cardiopulmonary pathology was excluded. Deconditioning was defined as the result of decreased physical activity, bed rest, disease, ageing or other causes. The term non-pathologic diagnosis was used for conclusions which don’t have an underlying illness such as deconditioning, obesity, dysregulated breathing mechanisms and patients where no explanation was found. Dutch law allows the use of electronic health records for research purposes under certain conditions. According to this legislation, neither obtaining informed consent from patients is obligatory for this type of observational studies containing no directly identifiable data (Dutch Civil Law, Article 7: 458). The conduct of the study was approved by the Leiden University Medical Center medical ethics committee. Methods were performed in accordance with relevant regulations and guidelines

### Statistical analysis

Two patient groups were compared: 1) patients with an integrated consultation (dyspnea clinic) and 2) patients with a non- integrated consultation, who were first seen by either the cardiologist or the pulmonologist on separate occasions without consultation between specialists. Baseline characteristics, diagnostics used, time to referral, diagnosis and medical conclusions were compared with unpaired t-tests for normally distributed variables, Mann-Whitney U tests for non-normally distributed variables and chi-square tests for categorical variables. Outcomes for referral and diagnostic times were adjusted for differences in baseline characteristics by multivariate linear regression analyses. Data was extracted using Microsoft Access (Microsoft Office 2013) and analyzed with SPSS Windows versions 23.0.0.2. (IBM Corp, Armonk NY). Graphics were made with GraphPad Prism 7.0.0. for Windows (GraphPad Software, La Jolla California USA).

## Results

**Figure 2:**
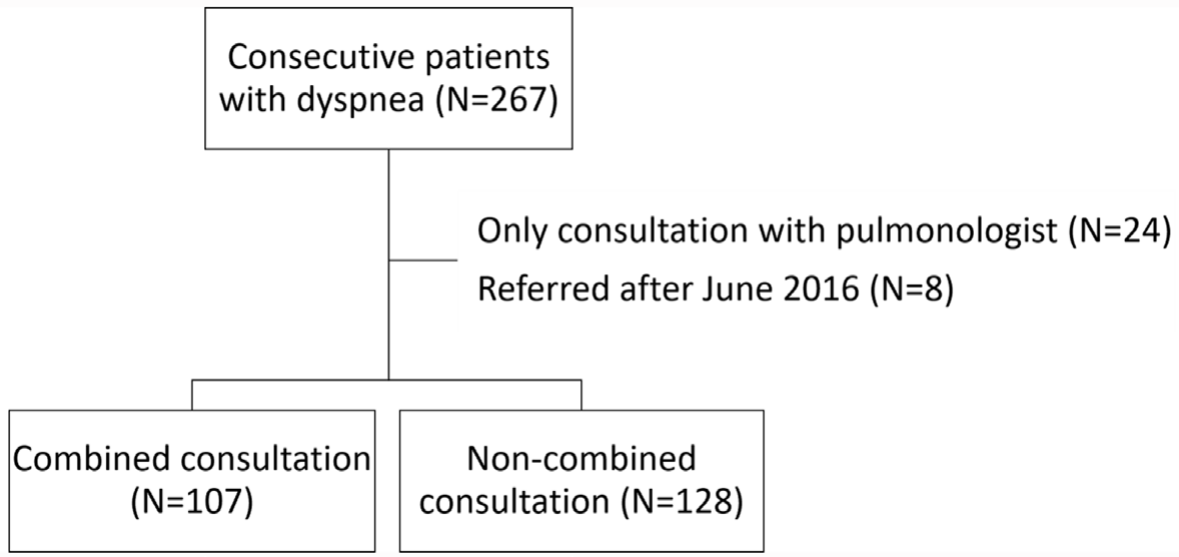
Flowchart of included patients.

In total 267 patients were included during the study period. 24 patients were excluded because they were only seen by a pulmonologist for a non-integrated consultation. Additionally, 8 patients were excluded because they were referred after June 2016 (Figure 2). For the analyses, 107 patients with an integrated consultation and 128 patients with non- integrated consultations were included.

### Baseline characteristics

Baseline characteristics of patients between integrated and non- integrated consultations were similar (Table 1). However, patients in the integrated consultation group less often had a extensive cardiovascular medical history than patients in the non- integrated group (30% vs. 56%, p ≤ 0.001).

**Table 1:** Baseline characteristics of study population (n = 235) attending the dyspnea clinic.

	Integrated consultation (N = 107)	Non-integrated consultations (N = 128)	P-value

Age in years	62 (14.4)	61 (16.1)	0.656
Male gender	48.6%	46.1%	0.793
BMI (kg/m^2^)	28.1 (5.0)	27.7 (5.5)	0.610
Smoke status			
Smoker	14 (13.1)	19 (14.8)	0.851
Former smoker	55 (51.4)	63 (49.2)	0.794
Non-smoker	38 (35.5	46 (35.9)	1.000
Medical history			
No history	4 (3.7)	4 (3.1)	1.000
Pulmonary	58 (54.2)	76 (59.4)	0.431
Mild cardiovascular^a^	29 (27.1)	15 (11.7)	0.004
Extensive cardiovascular^b^	32 (29.9)	72 (56.3)	0.000
Other	90 (84.1)	105 (82.0)	0.729
Medication usage			
No medication	10 (9.3)	13 (10.2)	1.000
Pulmonary	40 (37.4)	45 (35.2)	0.786
Cardiovasculair	61 (57.0)	87 (68.0)	0.103
Other	85 (79.4)	97 (75.8)	0.534

Variables are depicted as mean and standard deviation for continuous variables or as number and proportion for categorical variables. ^a^ Contains history of hypertension, asymptomatic vascular disease and/or hypercholesterolemia. ^b^ Contains history of ventricular dysfunction (systolic and diastolic), any arrhythmia, pacemaker implementation, conduction abnormalities, Angina Pectoris (AP), valvular disease, congenital heart disease, Transient Ischaemic Attack (TIA) or Cerebrovasculair accident (CVA), thrombosis, myocardial infarction, pulmonary embolism, Coronary artery bypass grafting (CABG), Percutaneous coronary intervention (PCI) with stents and peripheral vascular disease.

### Referral

Most patients (96/107, 90%) with a integrated consultation were referred by their general practitioner (GP); only 2 (2%) were referred by a cardiologist and 9 (8%) by other specialists. Most patients in the non- integrated consultation group were referred by the cardiologist (107/128, 84%), 9 (7%) were referred by their GP, 9 (7%) by other specialists and 3 (2%) by a pulmonologist.

### Diagnostic tests

The type and number of diagnostic tests per patient group is shown in Table 2. For most diagnostics, there was no difference in usage between the two groups. Only echocardiography was performed more often in the integrated consultation group (99% vs. 88%, p = 0.001) and a chest x-ray was performed more in the non- integrated consultation group (16% vs. 45%, p = 0.015).

**Table 2:** Diagnostics used in patients (n = 235) attending the dyspnea clinic.

Characteristic	Integrated consultation (N = 107)		Non- integrated consultation (N = 128)		P-value

	Yes	No	Yes	No	

Standard diagnostics^a^, N (%)					
Basic laboratory^b^	106 (99)	1 (1)	127 (99)	1 (1)	1.000
Electrocardiogram (ECG)	106 (99)	1 (1)	125 (98)	3 (2)	0.628
Echocardiography	106 (99)	1 (1)	113 (88)	15 (12)	0.001
Spirometry	105 (99)	2 (1)	127 (99)	1 (1)	0.593
Exercise ECG	69 (65)	38 (36)	75 (59)	53 (41)	0.420
Mean (SD) number of standard diagnostics per patient		4.6 (0.6)		4.4 (0.8)	0.057
Additional diagnostics N (%)					
Advanced laboratory^c^	29 (27)	78 (73)	43 (34)	85 (66)	0.321
Chest x-ray	31 (29)	76 (71)	57 (45)	71 (56)	0.015
Chest CT scan	17 (16)	90 (84)	27 (21)	101 (79)	0.320
Thoracic ultrasound	3 (3)	104 (97)	3 (2)	125 (98)	1.000
Ventilation perfusion scan	7 (7)	100 (94)	7 (6)	121 (95)	0.787
Histamine provocation test	10 (9)	97 (91)	9 (7)	119 (93)	0.632
Fractional exhaled nitric oxide (FeNO) test	7 (7)	100 (94)	8 (6)	120 (94)	1.000
Cardiopulmonary exercise test (CPET)	19 (18)	88 (82)	17 (13)	111 (87)	0.368
Sputum culture	1 (1)	106 (99)	1 (1)	127 (99)	1.000
Bronchoscopy	1 (1)	106 (99)	1 (1)	127 (99)	1.000
24 hour Holter-ECG	32 (30)	75 (70)	28 (22)	100 (78)	0.178
Supplementary echocardiography	5 (5)	102 (95)	1 (1)	127 (99)	0.095
Coronary CT angiography	15 (14)	92 (86)	12 (9)	116 (91)	0.307
Myoview	8 (8)	99 (93)	4 (3)	124 (97)	0.148
Dobutamine stress echocardiography	9 (8)	98 (92)	14 (11)	114 (89)	0.660
Coronary angiography	10 (9)	97 (91)	23 (18)	105 (82)	0.062
Thoracocentesis	1 (1)	106 (99)	2 (2)	126 (98)	1.000
Polysomnography	6 (6)	101 (94)	5 (4)	123 (96)	0.554
PET scan	0 (0)	107 (100)	2 (2)	126 (98)	0.502
Mean (SD) number of additional diagnostics per patient		2.0 (1.5)		2.1 (1.5)	0.644
Mean (SD) number of total diagnostics used per patient		6.6 (1.4)		6.5 (1.9)	0.724

^a^ Standard diagnostics when visiting the dyspnea clinic. ^b^ Basic laboratory includes hemoglobin-value (Hb) and renal function. ^c^ Advanced laboratory includes all other laboratory diagnostics. When the referring general practitioner performed laboratory only Hb and renal function was included for basic laboratory diagnostics, other laboratory diagnostics were not included for advanced diagnostics.

### Diagnostic time during the care trajectory

**Figure 3:**
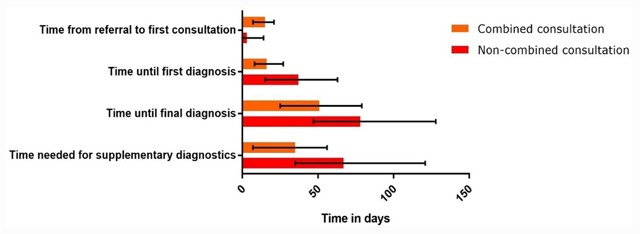
Diagnostic time during care trajectory. Time is depicted in median days with IQR.

Days between referral and first consultation (waiting time), referral and first diagnosis (time until first diagnosis), referral and final diagnosis (time until final diagnosis) and first consultation and final diagnosis (time needed for supplementary diagnostics) is shown Figure 3. Waiting time was shorter in the non- integrated consultation group (median 15 days vs. 3 days, p < 0.001). Time until first diagnosis, final diagnosis and time needed for diagnostics was shorter in the integrated consultation group (median 16 days vs. 37 days, p < 0.001; 51 days vs. 78 days, p < 0.001; 35 days vs. 67 days, p < 0.001). These differences in diagnostic times remained similar after correction for baseline differences in the extensiveness of cardiovascular history with multivariate regression analysis.

### Medical conclusions

The medical conclusions for each patient group are displayed in Table 3. There were no statistical significant differences in most medical conclusions between the integrated and non- integrated consultation group. Only ‘hypertension’ and ‘no explanation’ were diagnosed more often in integrated consultation group (14% vs 3%, p = 0.003 and 19% vs. 6%, p = 0.002 respectively). Chronotropic insufficiency was diagnosed more often in the non- integrated consultation group (8% vs. 16%, p = 0.046). There was no difference in the number of conclusions between both groups (mean 1.5 vs. 1.6, p = 0.214).

**Table 3:** Medical conclusions made in the integrated and non-integrated consultation groups.

Characteristic, N(%)	Integrated consultation (N = 107)	Non-integrated consultation (N = 128)	P-value

Symptomatic cardiovascular disease	30 (28.0)	60 (46.9)	0.005
Angina Pectoris	10 (9.3)	13 (10.2)	1.000
Chronotropic insufficiency^a^	8 (7.5)	21 (16.4)	0.046
Other arrhythmia’s	7 (6.5)	8 (6.3)	1.000
Valvular disease	6 (5.6)	10 (7.8)	0.607
Ventricular dysfunction; systolic; symptomatic	2 (1.9)	9 (7.0)	0.071
Ventricular dysfunction; diastolic; symptomatic	1 (0.9)	6 (4.7)	0.130
Secondary pulmonary hypertension	2 (1.9)	3 (2.3)	1.000
Symptomatic dyspnea due to anemia	2 (1.9)	5 (3.9)	0.459
Atrial fibrillation	2 (1.9)	4 (3.1)	0.691
Pulmonary embolism	0 (0.0)	4 (3.1)	0.128
Congenital heart disease	0 (0.0)	2 (1.6)	0.502
Conduction abnormalities	0 (0.0)	0 (0.0)	–
Asymptomatic cardiovascular disease	21 (19.6)	15 (11.7)	0.104
Hypertension	15 (14.0)	4 (3.1)	0.003
Asymptomatic vascular disease	5 (4.7)	11 (8.6)	0.302
Ventricular dysfunction; systolic; asymptomatic	2 (1.9)	0 (0.0)	0.206
Ventricular dysfunction; diastolic; asymptomatic	1 (0.9)	1 (0.8)	1.000
Pulmonary disease	47 (43.9)	67 (52.3)	0.238
COPD	22 (20.6)	31 (24.2)	0.534
Asthma	9 (8.4)	21 (16.4)	0.079
Combination of asthma and COPD	3 (2.8)	3 (2.3)	1.000
Exacerbation of known asthma or COPD	3 (2.8)	4 (3.1)	1.000
Aspecific bronchial hyper reactivity	5 (4.7)	3 (2.3)	0.474
OSAS	3 (2.8)	5 (3.9)	0.731
Post pulmonary embolism syndrome	0 (0.0)	3 (2.3)	0.253
Interstitial lung disease	3 (2.8)	0 (0.0)	0.093
Thoracic deformities	1 (0.9)	0 (0.0)	0.455
Sarcoidosis	0 (0.0)	0 (0.0)	–
Non-pathologic diagnosis	51 (48)	56 (44)	0.600
Deconditioning^b^	18 (16.8)	29 (22.7)	0.326
Dysregulated breathing mechanisms	15 (14.0)	17 (13.3)	1.000
Obesity	13 (12.1)	24 (18.8)	0.209
No explanation	20 (18.7)	7 (5.5)	0.002
Spontaneous improvement before analysis	1 (10.9)	1 (0.8)	1.000
Malignancies	3 (2.8)	2 (1.6)	0.662
Lung cancer	1 (0.9)	1 (0.8)	1.000
Metastatic disease	2 (1.9)	2 (1.6)	1.000
Other explanations	10 (9.3)	4 (3.1)	0.055
Mean (SD) number of conclusions per patient	1.5 (0.7)	1.6 (0.7)	0.21

Values are in n (%) unless stated otherwise. Patients can have more than one conclusion. COPD, chronic obstructive pulmonary disease. OSAS, obstructive sleep apnea syndrome. ^a^ Inability of the heart to increase its rate to compensate for increased activity or demand. ^b^ Result of decreased physical activity, bed rest, disease, aging or other causes.

## Discussion

The aim of this study was to evaluate whether an integrated care dyspnea clinic, in which patients are evaluated in an integrated consultation of a cardiologist and pulmonologist, is more efficient than regular care. The main findings were as follows. First, the baseline characteristics of our study groups were largely similar, but the non- integrated consultation group had a more complex cardiovascular history than the integrated group. Second, the median time needed for diagnostic workup was almost one month shorter for the integrated consultation group. Third, the majority of medical conclusions made in the dyspnea clinic was similar between the integrated and non- integrated consultation groups.

### Interpretation and comparison with previous literature

Previous studies have shown a high proportion of cardiovascular comorbidity in pulmonary patients and vice versa. In line with previous authors, we see an urgent need for integrated diagnostic workup for patients with dyspnea [13]. We are one of the first to shed scientific light on the efficiency gain of a dyspnea clinic. A previous study suggested improved care with a dyspnea clinic, but did not incorporate a control group [10]. Patients in our control group (non- integrated consultation) had a more complex history of cardiovascular disease compared with patients in the integrated group. This difference is most likely due to the fact that patients in the non- integrated consultation group included patients who were already under regular treatment of a cardiologist. This difference in extensiveness of cardiovascular medical history may also have caused hypertension to be diagnosed more often in the integrated consultation group, because patients in the non- integrated consultation group already had treatment for their blood pressure. ‘No explanation’ was diagnosed more often in the integrated consultation group. The reason for this might be that a discussion between both specialists about the final diagnosis was part of the integrated consultation care process. During these discussions, both pulmonary and cardiovascular causes of dyspnea can be excluded with more certainty and a ‘no explanation’ conclusion is set more often instead of referring to the other specialist again.

There were small differences in diagnostics used between our integrated and non- integrated consultation groups. Echocardiograms were used more often in the integrated consultation group. An explanation for this might be that patients in the non- integrated group an echocardiography was already preformed during regular cardiology consultation, before the date of referral to the dyspnea clinic.

We found that the waiting time was shorter in the non-integrated group. One of the reasons could be that this patient group included patients already under treatment by the cardiologist. For these patients the date of referral was often equal to the date of consultation with the pulmonologist, making the waiting time equal to zero days. This not only influences waiting time but also could have reduced the time until first diagnosis and time until final diagnosis in the non-integrated consultation group. Still, the median time for these outcomes was shorter for the integrated consultation group. So, we expect the differences in time of diagnostic work-up to be even an underestimation.

In our patient cohort, a non-pathologic diagnosis was concluded often. This rate of non-pathologic diagnoses reflects regular care and is in line with previous studies in which no diagnosis for the dyspnea was concluded in 9–19% of patients [4]. Another study found no explanation for dyspnea in 24% of the patients [15]. However, only heart failure and pulmonary causes were analyzed as outcomes in this study, and no other explanations for dyspnea were analyzed, whereas other non-pathologic explanations can be very valid in explaining dyspnea, such as obesity related changes in work of breathing, deconditioning, or dysregulation of breathing, after excluding other cardiac and pulmonary diagnoses.

In our study, we selected the diagnostic time and number of diagnostics used as efficiency measures. We realize that numerous other efficiency frameworks and measures on patient level or health care system level are available and could have been used to evaluate the dyspnea clinic [14]. However, we think that ‘diagnostic time’ and ‘number of diagnostic tests’ are very important efficiency measures from the patient perspective and are very relevant to physicians in primary and secondary care. Still, future studies could focus on other measures and models of health care efficiency.

### Strengths and limitations

A strength of this study is that patients were listed in the electronic medical record by the attending pulmonologist consecutively. This guarantees that all patients, and not only less complex patients, were included and thus excludes major selection bias. Another strength of the present study is that multiple conclusions per patient were allowed. This resembles clinical practice, because it is known that dyspnea can be caused by multiple conditions.

The most important limitation of the study seems the origin of the patients in the non-integrated care group. We included patients who were referred to, or where under control of a cardiologist. Ideally, we would have prospectively included patients from primary care referred for dyspnea and randomized the integrated and non-integrated care groups. However, we still think our observational study and control group is valid and answers a relevant clinical question for three reasons. First, in regular non-integrated care, patients with dyspnea are referred to either a cardiologist or a pulmonologist, instead of the two specialists together. In our experience, because of the time needed for diagnostics by each specialism and cross-referral, regular non-integrated care will generally take a longer time to final diagnosis than integrated care. In our opinion, the comparison of the two patient groups in our study reflects this difference in diagnostic time in every day clinical practice. Second, by including patients already referred to a cardiologist as a control group, the diagnostic time in this group is probably shorter than a patient group with dyspnea from general practice. Still, the median time to diagnosis was shorter for the integrated consultation group. So, with the use of cardiology controls, we expect the differences in time to final diagnosis to be even an underestimation. Third, a confounding might have been introduced by selecting patients from a cardiology clinic as a control group. However, the differences in diagnostic times remained similar after correction for baseline differences between groups with multivariate regression analysis. In summary, although our control group was not the ideal theoretical control group, the results of our study are still valid, answer a relevant clinical question and are probably an underestimation of the differences we would have observed by selecting a control group from primary care.

Another limitation is that all data were collected from only one hospital (Leiden University Medical Center). Because the Leiden University Medical Center is a tertiary line center it is possible the diagnostic time during the care trajectory is longer here than in a secondary line center. Therefore, the results found might not be applicable to other dyspnea clinics connected to less specialized hospitals. Furthermore, the duration of the dyspnea was not registered in our study and therefore it is impossible to say if there is a difference in chronicity between our groups. Finally, we assume that a faster diagnosis is beneficial for patients and reflects better efficiency. However, we did not measure patient satisfaction and health care costs. These measures would be valuable to add in future studies.

### Clinical implications and future research

This study is, to our knowledge, the first assessing efficiency of an integrated care dyspnea clinic. This study shows that diagnostic time can be substantially reduced with a dyspnea clinic. This is especially important for patients, who spend less time in uncertainty about possible serious disease and to allow non-pathologic explanations for dyspnea due to effective consultation between cardiologist and pulmonologist. Future studies could include other patient populations, for example patients referred from general practice to cardiologist or pulmonologist for the non-integrated group and compare this with patients directly referred from general practitioners to a dyspnea clinic. Preferably these studies should be multicenter prospective (randomized) studies to improve generalizability. Furthermore, integrated care for patients with dyspnea could also focus on mental and systemic consequences. This is an important topic for the development and innovation of integrated care for dyspnea patients in the future.

Finally, future studies could assess patient and doctor satisfaction and costs with this new care model.

## Conclusions

Our results show that patients evaluated in the integrated care dyspnea clinic seem to be diagnosed almost one month faster than patients in regular care without an increase in the number of diagnostics needed and without affecting the type of medical conclusions made. This is especially important for patients, who spend less time in uncertainty about possible serious disease. This study supports the start of a dyspnea clinic as an efficient way to provide integrated care to patients with dyspnea.
